# A peer coach intervention in childcare centres enhances early childhood physical activity: The Active Early Learning (AEL) cluster randomised controlled trial

**DOI:** 10.1186/s12966-021-01101-2

**Published:** 2021-03-16

**Authors:** R. M. Telford, L. S. Olive, R. D. Telford

**Affiliations:** 1 Research Institute for Sport and Exercise, University of Canberra, Canberra, ACT Australia; 2 School of Psychology, Deakin University, Geelong, Victoria Australia; 3 Centre for Social and Early Emotional Development, Faculty of Health, Deakin University, Geelong, Victoria Australia; 4 IMPACT, The Institute for Mental and Physical Health and Clinical Translation, Faculty of Health, Deakin University, Geelong, Victoria Australia

**Keywords:** Preschool, Childcare, Early learning, Physical activity, Peer coach, Professional development, Physical activity intervention, Physical activity program, RCT

## Abstract

**Background:**

As numbers of children and time spent in childcare centres increase, so does the potential influence of these centres on early childhood physical activity (PA). However, previous reports indicate little success of interventions aimed at improving PA. The Active Early Learning (AEL) program is a multi-component pragmatic intervention designed to imbed PA into the daily curriculum. Delivered by childcare centre staff, it is directed and supported by a peer coach who works across a network of centres. The objective of the study is to investigate the effect of the AEL program on children’s PA.

**Methods:**

Fifteen childcare centres (8 intervention, 7 control centres; 314 children, 180 boys, 4.3y ± 0.4) participated in a 22-week stratified cluster randomised controlled trial. To be eligible to participate, centres needed to have ≥15 preschool children aged 3 to 5-years. The primary outcome was PA measured by accelerometer (Actigraph GT3X) during childcare centre hours over a 3-day period, calculated in min/h of Total PA and moderate-to-vigorous PA (MVPA). The effect of the intervention was evaluated using linear mixed models adjusted for age, sex, accelerometer wear time and centre clustering.

**Results:**

There was an intervention effect for Total PA (+ 4.06 min/h, 95% CI [2.66 to 5.47], *p* < .001) and MVPA (+ 2.33 min/h, 95% CI [1.31 to 3.34] p < .001). On average, a child taking part in the intervention attending a childcare centre from 8 am to 3 pm performed 28 min more Total PA and 16 min more MVPA per day than children receiving usual practice care.

**Conclusion:**

In contrast with the findings of previous pragmatic trials in early childcare centres, this study shows that a peer-coach facilitated program, focussed on integrating PA into the daily childcare routine, can elicit increases in preschool children’s PA of practical as well as statistical significance.

**Trial registration:**

Australian New Zealand Clinical Trials registry: ACTRN12619000638134. Registered 30/04/2019.

## Background

Physical activity plays a central role in children’s health and wellbeing and is associated with a range of psychosocial, cognitive and physical health outcomes [1]. There is also increasing evidence that physical activity levels track into later stages of life [2], with early childhood emerging as a critical time for the promotion of healthy lifestyle behaviours [3]. Childcare centres are an ideal setting to address physical activity behaviour across the community because they are attended by a large proportion of preschool aged children. For example, in Australia, 85% of children attend non-compulsory preschool, of which 50% attend a preschool program for 15 or more hours per week at a childcare centre [4].

Physical activity interventions in childcare settings have been the topic of several meta analytic reviews [5–7], systematic reviews [5, 8] and commentaries [9] from which there is general consensus that interventions specifically targeting physical activity may evoke a small to moderate effect. Despite insufficient current evidence to describe any specific approach most likely to succeed, authors have outlined intervention characteristics that appear to promote success. These include: structured activities easily incorporated into the daily ‘routine’ [6, 7]; professional development of educators and carers [5, 8], as well as alternate methods to traditional face-to-face professional development, such as mentoring, and coaching [9]; specific targeting of the group of interest [7]; pragmatic considerations that suggest “real world” application [6]; and appropriate theory in the design process [8]. Of particular interest was the conclusion drawn in a recent meta-analysis [6] that not one pragmatic intervention in childcare settings had been successful in improving physical activity.

Our approach and evaluation, novel to childcare settings, is to provide childcare centre educators with in-centre professional development from a peer coach. Peer coaching can include planning, teaching, modelling, and practising new skills, direct observation of the implementation of target practices, and performance feedback [10]. Deploying just the one peer coach across multiple centres not only provides tailored support and feedback to assist educators to conduct the physical activities themselves, but also promotes a system of economic and administrative efficiency, and so sustainability. Indeed, a peer coach used in this manner has previously been found to be successful in improving physical activity levels in primary school-aged children [11].

The aim of this study was to evaluate the effect of a multisite and pragmatic peer coach-based intervention on the level of physical activity of the children attending childcare centres.

## Method

### Study design

**Fig. 1:**
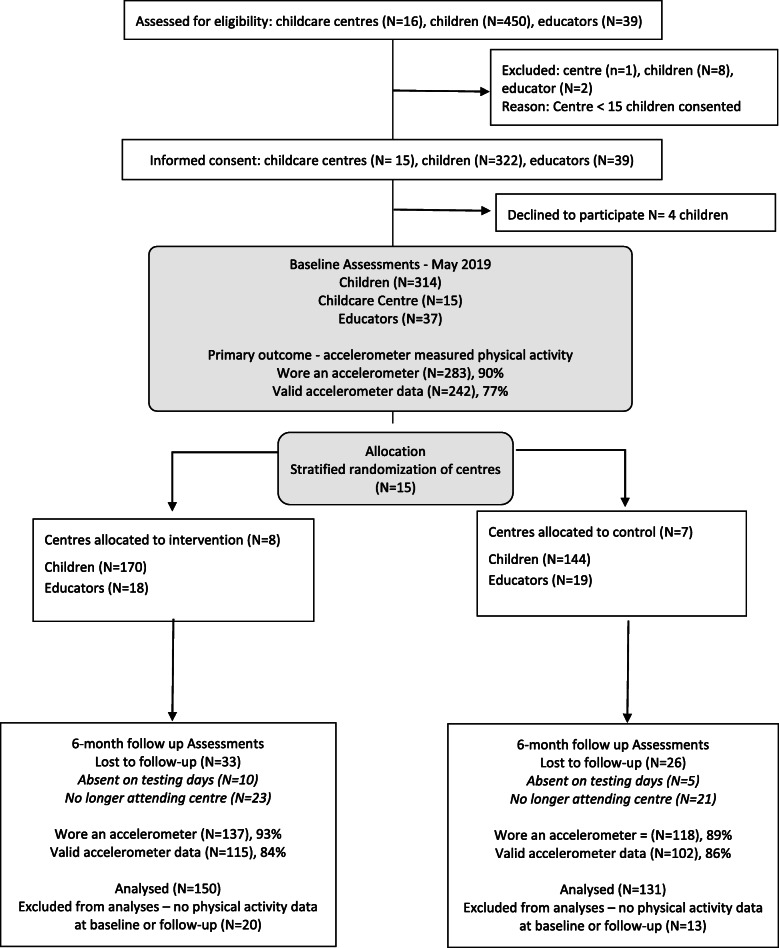
Consort flow diagram showing the progress of participants through the randomised controlled trial

Conducted over a 6-month period, the Active Early Learning (AEL) intervention was a cluster randomised controlled trial (RCT) in 16 childcare centres, each individually owned by one of 3 private companies. Following consent by the childcare centre owners, these sixteen centres were invited to participate in the study. Eligibility criteria for a centre to participate included having a minimum attendance of 15 preschool children between the ages of 3 to 5-years. The study followed the Consolidated Standards of Reporting Trials (CONSORT) Statement with Extension to Cluster Randomised Trials [12] and was registered with the Australian New Zealand Clinical Trials Registry (ACTRN12619000638134) and approved by the University of Canberra Human Research Ethics Committee (No:1853). The general study design is depicted in the CONSORT diagram Fig. 1.

### Randomisation and allocation

Childcare centres in this stratified cluster RCT were assigned to trial arms such that centre-level covariates (socioeconomic status [SES], National Quality Rating (NQR) and geographic location) were balanced. Firstly, centres were stratified according to higher SES > 1000 and lower SES < 1000, using the Australian Bureau of Statistics Socio-Economic Indexes for Areas (SEIFA) index [13]. These strata were then split according to the NQR of each centre, a government quality rating system for childcare centres [14]. The NQR involves an assessment of 7 categories of centre quality: educational program and practice, physical environment, health and safety, staffing arrangements, relationships with children, collaborative partnerships and governance and leadership. Each category is rated 1 to 3 permitting a maximum score of 21. With the mean NQR score and range for the centres in the study being 14.1 (9–18), the centres were stratified according to rating lower with an NQR < 14 or higher with an NQR > 14. This produced four groups: (1) Higher SES/higher NQR, (2) Higher SES/lower NQR, (3) Lower SES/lower NQR, and (4) Lower SES/higher NQR. Following baseline data collection, centres from each stratum were allocated to the intervention or control arm using a computer-generated randomization procedure, conducted by an independent researcher.

### Study setting

The childcare centres are located in New South Wales and southern Queensland in Australia. All centres are privately owned and provide all-day or part-time care for children aged 6 months to 5 years, and they provide a preschool education program for children (typically 3 to 5 years of age) prior to commencing primary school. The curriculum is guided by the Early Years Learning Framework developed by The Council of Australian Governments, and as outlined above are assessed against the National Quality Framework (NQF) which provides a national approach to regulation, assessment and quality improvement for early childhood education and care settings [14]. Children in this age group have their own room, and their supervision must include an early childhood teacher with university degree qualification; and all other educators must be working toward an approved certificate course in early childcare with a minimum educator to preschool child ratio of 1:11. In the current study, the average number of preschool children was similar across the centres (mean = 29, range = 20 to 35), and the mean NQR score of the centres (14.1) was lower than the mean of all childcare services in Australia (16.4 ± SD 5.7). Centres allocated to the intervention group received the AEL program and control centres continued with their usual practice.

### The intervention

#### The AEL coach

**Fig. 2:**
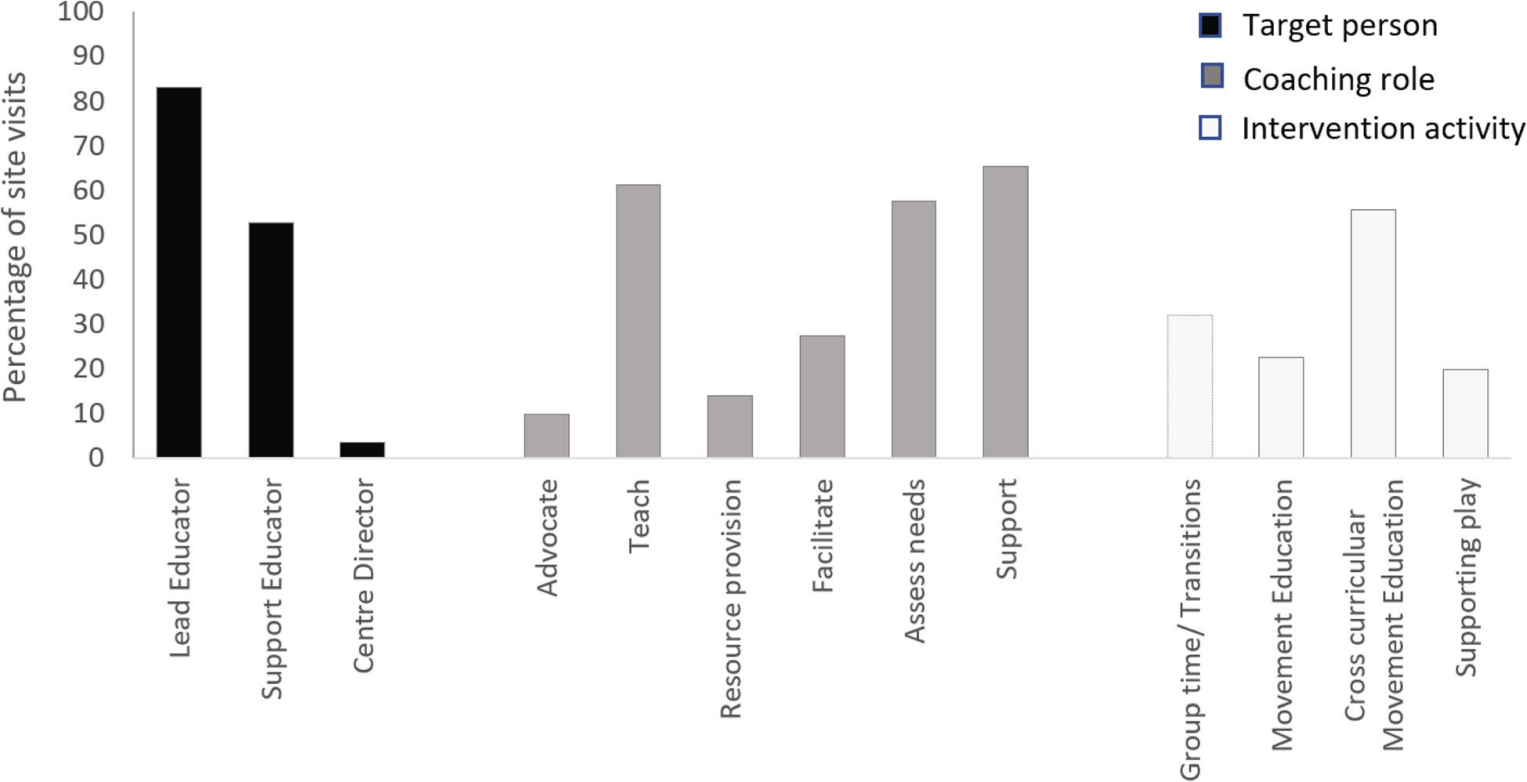
Relative proportion of AEL coach visits, classified by target staff member, coaching role and intervention activity

The centrepiece of the intervention is the appointment of a peer coach (the AEL coach), whose role is to introduce program components to the educators sequentially during a weekly onsite visit. Consistent with a pragmatic approach, we employed one AEL coach who resided within the general geographical area of the childcare centres. The position was advertised on an employment website with qualifications deliberately outlined in broad terms. These were listed as experience in early childhood education or physical activity, a desire to work with children and childcare workers, and strong personal communication skills. The level of remuneration was set accordingly at an industry rate considered viable by centre operators for future scaled up implementation. The successful applicant, who had 5 years’ experience delivering children’s physical activity programs in childcare and not-for-profit settings, as well as a qualification in social work, participated in 4 workshops delivered by the research staff. The topics covered included: a) the AEL program background, philosophy and objectives; b) physical literacy and its relationship with physical activity; c) opportunities for the development of physical literacy and improving physical activity within the childcare centre setting and curriculum; and d) peer coaching strategies and their application with childcare educators. These peer coaching strategies can be broadly categorised as: a) advocating (the importance of physical activity and physical literacy); b) teaching (how to plan and deliver program activities); (c) resource provision (activity plans and ideas); c) facilitating (meeting curricular requirements through AEL activities); d) supporting (educator encouragement and motivation); and e) assessing (progress, potential barriers, problem solving). The frequency of each strategy used by the coach is shown in Fig. 2.

#### Theoretical framework

The role of the AEL coach is informed by Vygotsky’s Social Development Theory (SDT), which argues that social interaction precedes development [15]. Using SDT to inform the design and implementation of teacher professional development has previously been discussed and recommended [16]. SDT places importance on the connections between people (the AEL coach and childcare staff) and the sociocultural context (childcare setting) in which they act and interact in shared experiences (the AEL program of activities). SDT states the important role of a ‘More Knowledgeable Other’ who imparts knowledge on a learner. This person refers to anyone who has a better understanding or a higher ability level than the learner, with respect to a task, process, or concept. The ‘More Knowledgeable Other’ is normally thought of as being a teacher, coach, older adult or peer, and in the case of the current study, is the AEL coach. We apply SDT to inform the relationship between the AEL coach and childcare educator, as well as the relationship between childcare educator and the children under their care.

The design of the intervention was informed by the concept of physical literacy [17], which acknowledges the interplay between an individual’s level of physical activity and their level of motivation, confidence, physical competence and knowledge. Activities were therefore selected to prioritise enjoyment while developing movement competency and confidence, rather than solely focussing on increasing physical activity. Accompanying AEL resources, provided in the form of printed cards, described how each activity contributed to the development of physical literacy. Contributing elements were categorised within the four domains of physical literacy set out by the Australian government [18]: the physical domain (e.g. throwing and catching); the psychological domain (e.g. enjoyment); the social domain (e.g. teamwork); and the cognitive domain (e.g. decision making).

#### Development

A fundamental consideration was to develop a pragmatic intervention; in other words, one designed for sustainable operation under “real world” childcare centre conditions. An Intervention Mapping Planning Framework [19] was employed to inform AEL intervention design; a 6-step systematic process for decision making, implementation and evaluation. The process followed was to: a) examine past literature and consult with experienced researchers and practitioners to better understand behavioural and environmental determinants of children’s physical activity in childcare settings; b) identify desired program outcomes, select determinants of behavioural and environmental change and create intervention objectives; c) identify theories providing insight as to how the objectives could be operationalised in the childcare setting; d) decide on intervention components and integrate them into the AEL intervention; e) develop an implementation strategy to embed the program into the curriculum and daily routine of the childcare centres; and f) plan the evaluation of the program implementation and effectiveness. With the pragmatic qualities of our intervention at the forefront of our considerations, and to facilitate steps b) to e), we conducted four focus groups with childcare staff to seek their views on physical activity, physical development and professional development. We also consulted the centre owners throughout the design process to ensure the proposed implementation method was considered practically and financially realistic.

#### Description

The intervention mapping process to increase physically active experiences into the childcare centre daily routine identified four opportunities. As described in Table 1, along with the sequence and period of their introduction, these were classified as: group/mat time and transitions, movement education, cross-curricular movement education, and encouraging challenging free play.

**Table 1:** The AEL program components, frequency and description

AEL program components^a^	Week of introduced components	Description and typical duration
1. Group/mat time and Transitions	1 to 4	Group/Mat time: daily periods when children are gathered together, movement experiences with a focus on fundamental movement skill; 5 to 20 min. Example: Educator holds up pictures of animals or objects and children explore associated movements, such as frog’s jump, rocks’ stillness. Transitions: periods between different centre events e.g. arrival at the centre, moving from inside to outside, or from group time to meal tables with a focus on movement creativity and exploration; 1 to 5 min. Example: Children mimic their favourite animal as they move from mat time to hand washing.
2. Movement education	5 to 8	Educator guided individual or group physical activity challenges which may involve equipment, sports and games; 10–20 min. Example: Games incorporating balls and other equipment; obstacle course challenges
3. Cross-curricular movement education	9 to 12	Activities complementing the day’s curriculum learning theme; usually integrating a story and book reading; 20–30 min. Example: Children are read a story about the circus. The educator guides children to discuss and perform circus movements such as walking along a line of tape (tight rope walking), exploring bean bags (juggling).
4. Encourage challenging free play	13 to 16	Educator enhancement of free-time active play to promote exploration and opportunities to develop confidence and risk assessment skill with challenging play; 10–30 min. Challenging play in this study was communicated to educators as using movement to explore boundaries and test children’s limits. Example: Coach and educators discuss and then encourage challenging play using a selected piece of outdoor equipment (such as a balance beam or slide) while taking safety precautions.

^a^Intervention components 1 to 4 are re-introduced concurrently by childcare educators during weeks 17 to 22, with daily inclusion of components 1 to 2 (or 3)

Of the 22-week intervention, each component was introduced and developed over 4 weeks, with all 4 components incorporated into the daily schedule during the following 6 weeks. The professional development provided by the AEL coach for the educators included guidance on introducing and conducting the activities. This occurred during a weekly “in-class” site visit which involved coach-educator reflection on the degree of success of the previous weeks’ activities followed by the planning of activities for the coming week.

Coach-educator interaction occurred during class time during a prearranged 3-h window of opportunity either in the morning or afternoon. The coach exercised a large degree of flexibility to accommodate the dynamic childcare environment, working with the educators as time became available, visiting two centres per day from Monday to Thursday. Friday was a dedicated planning day for the coach to organise the following weeks site visit and to plan how to tailor the activities into the learning themes at the centres. For example, if the weekly theme was “colours”, the coach devised ways in which colours could be incorporated into that week’s physical activities. During the focus groups, the educators indicated a preference for physical print-outs rather than online resources. Consequently, printed summaries of the program activities were provided prior to each site visit outlining preparation and equipment requirements. As previously mentioned, these also included, in simple terms, the physical, social and psychological objectives aligned with each activity. While this resource was provided each week, educators were also encouraged to be creative, and modify activities based on their experience and preferences.

### Measurements

Collection of baseline data occurred over 3.5 weeks in May 2019 with the post-intervention measures occurring for the same duration in November/December of the same year.

#### Demographics and participant characteristics

Children’s height and weight were measured using a portable stadiometer (Wedderburn, Model no: WM602) and digital scales (A&D, Model no: UC321). Date of birth, sex and attendance were recorded, and educators completed an online survey requesting their age, level of education and years of childcare experience. The SEIFA index was used to estimate the socio-economic status of the childcare centre location.

#### Physical activity

Children wore accelerometers (Actigraph GT3x, Pensacola, FL, USA) on a waist belt for 3 consecutive days during childcare hours. Delegated educators at each centre were provided with written and verbal instruction and a demonstration of how to fit children with the accelerometers. Over 3 days, as each child arrived at a centre, the educator fitted the waist belt and removed the belt at time of departure. The physical activity outcome variables were total physical activity (Total PA) and moderate-to-vigorous physical activity (MVPA) measured as minutes per hour. Activity cut points were set for light activity (800–1679) and MVPA (above 1680), based on a previous calibration study for preschool children [20]. Total PA was the combination of light activity and MVPA. Using an epoch length of 15 s, data were included for analyses if there were ≥ 3 or more hours of valid wear time after screening for non-wear periods of ≥20mins. The selection of physical activity outcome variables, cut points and validation criteria were based on a previous study in preschool children [21].

#### Process measures

To assess implementation fidelity, and to summarise process measures, data were extracted from a daily online logbook maintained by the AEL coach. This included day by day records of the site visits, the type of activity, coaching strategy and details of educator progress. Lead Educators at each centre were asked to maintain a record of intervention activities on a wall chart provided by the AEL coach. To indicate educator fidelity, a custom rubric was created to assess the quality and frequency of the implemented activities; these being completed by a researcher using direct observation of educator practice followed by an interview.

### Statistical analysis

Independent sample t-tests were used to examine differences in the intervention and control groups at baseline. To investigate the effect of the intervention on physical activity, we fitted a Linear Mixed Model using the R Lme4 package [22] with Physical Activity as the dependent variable, Group-by-Time (i.e. intervention effect), Sex, Age and Accelerometer Wear Time as fixed effects, and Centre and Participants as random effects. Visual inspection of quantile-quantile plots and plots of fitted values versus the standardized residuals did not reveal any obvious deviations from homoscedasticity or normality. We performed significance testing using Type II F tests with Kenward-Rogers degrees of freedom approximation, and we report results as estimated mean effects with 95% confidence intervals. Our analysis was conducted using an intent-to-treat approach, and therefore included all randomised participants and all available data at each time point.

## Results

### Sample

Figure 1 shows the CONSORT flow diagram. Sixteen centres and 450 children were assessed for eligibility. Parents provided consent for 322 children (72% consent rate). Of the 16 centres, one centre had low enrolment numbers of preschool children (*n* = 8) and was excluded from the study. As a result, 314 children and 15 centres took part in the study.

Table 2 shows the baseline measurements of the participants, educators and centres. A higher proportion of boys (57%) than girls participated in the study and there was a higher proportion of girls in the intervention compared to the control arm (45% vs 40%). The results from the independent sample t-tests indicated there were no baseline differences in participant characteristics between the intervention and control groups. Of the 314 participants, 283 wore an accelerometer at baseline from which 242 (88%) returned valid data. At follow-up, 255 participants wore an accelerometer and 85% returned valid data. In the study jurisdiction, childcare staff are required by law to meet minimum standards of qualification and education, and those characteristics of the control and intervention groups were essentially the same. There were no significant group differences in educator age, SES or NQR.

**Table 2:** Pre- and post-intervention characteristics (unadjusted raw data) of the children, educators, and centres

	Intervention		Control	
	Pre	Post	Pre	Post
**N: Children**	170	147	144	123
**N: Boys**	93	80	87	73
**N: Girls**	77	67	57	50
**Age, years, mean (SD)**	4.3 (0.4)	4.8 (0.4)	4.3 (0.5)	4.9 (0.4)
**BMI**	16.4 (1.6)	16.4 (1.7)	16.2 (1.3)	16.1 (1.3)
**Centre attendance, days/wk**	3.6 (1.2)	3.6 (1.1)	3.3 (1.1)	3.2 (1.1)
**Valid accelerometer days**	2.0 (0.9)	2.2 (0.9)	2.1 (0.8)	2.3 (1.0)
**Accelerometer wear time, min/d**	406.3 (95.5)	340.8 (59.8)	414.7 (92.7)	336.4 (93.4)
**Total PA, min/h**	17.51 (4.54)	18.0 (4.6)	18.3 (5.1)	14.3 (5.6)
**MVPA, min/h**	9.2 (3.4)	9.4 (3.5)	9.6 (3.7)	7.3 (3.7)
**N who received accelerometer**	151	137	132	118
**N who returned valid data**	124 (83%)	115 (84%)	118 (89%)	102 (86%)
**N: lead educators**	8		7	
**N: support educators**	10		12	
**Educator age, years (mean, SD)**	30.6 (12.2)		29.8 (3.9)	
**N: educators with Cert III, Early Child Ed**	5		4	
**N: educators with Dip Early Child Ed**	5		8	
**N: educators with (or studying) University degree**	8		7	
**Childcare centre SES, SEIFA mean decile rank (range)**	7.5 (3–10)		7.3 (2–10)	
**Childcare centre National Quality Rating, mean score (range)**	14.1 (9–18)		13.8 (12–17)	

### Process measures

The frequency of coach visits was calculated from the coach logbook. The coach completed 164 out of a possible 176 site visits (93% completion of intended weekly visits); visiting each centre on average 21 times (range 18–22) from 22 possible weeks. Reasons for the coach missing a session were: sickness (*N* = 4), centre cancellation (N = 4) and public holiday (N = 4). As shown in Fig. 2, the coach worked mostly with the lead educator and support educators during the site visits, and most of her time was spent teaching and supporting educators to conduct the activities, along with assessing and addressing any emerging barriers.

From the rubrics designed to assess the frequency of intervention activities delivered by childcare staff, on average per week there were: 4 group time sessions (range 3 to 5), 5 transition activities (all educators reported 5) and 3 movement education or movement education extensions activities (range 1 to 4). There was no assessment of the frequency with which educators informally encouraged challenging free play.

### Intervention effects on physical activity

Table 3 shows the linear mixed model analysis for the dependent (response) variable Total PA and MVPA, expressed as minutes per hour. The Group by Time (intervention) effect on Total PA was 4.06 (95% CI [2.66–5.47], *p* < .001), indicating that for every hour a child spends at the childcare centre, those in the intervention were active for 4.06 min more than the control group. Based on an average attendance of 7 h per day, this effect is in the order of 28 min of Total PA per day.

**Table 3:** Linear mixed model analyses of the intervention effect on Total Physical activity and Moderate to Vigorous Physical activity

	Model 1 Total Physical Activity (min/h)			Model 2 Moderate to Vigorous Physical Activity (min/h)		
	*Estimates*	*CI*	*p*	*Estimates*	*CI*	*p*
***Fixed Effects***						
Intercept	8.75	3.45–14.04	0.001	2.98	−0.90 – 6.87	0.132
Time [POST]	−2.30	−3.54 – −1.05	< 0.001	−1.43	− 2.33 – −0.52	0.002
Group [Intervention]	−0.23	−2.32 – 1.87	0.833	−0.11	−1.54 – 1.31	0.875
Age	0.14	−0.95 – 1.22	0.808	0.32	−0.49 – 1.12	0.437
Sex [Boy]	2.75	1.83–3.67	< 0.001	2.05	1.37–2.73	< 0.001
Wear-time	0.02	0.01–0.02	< 0.001	0.01	0.01–0.01	< 0.001
Group by Time [POST]	4.06	2.66–5.47	< 0.001	2.33	1.31–3.34	< 0.001
***Random Effects***^a^						
Mean square variance σ^2^	13.29	3.65		6.84	2.61	
Between-participant variance	5.15	2.27		3.13	1.77	
Between-centre variance	3.07	1.75		1.32	1.15	
Intraclass Correlation Coefficient (ICC)	0.38			0.39		
N: Centres	15			15		
N: Participants	281			281		
N: Observations	451			451		

^a^ Random fffects are shown as estimates and standard errors

For MVPA, the intervention effect was 2.33 min/h, (95% CI [1.31–3.34], *p* < .001), indicating that for every hour a child spends at the childcare centre, those in the intervention performed 2.33 min more MVPA than the control group. Based on an average attendance of 7 h per day at the centre, this effect is in the order of 16 min of MVPA per day.

Total PA and MVPA models were adjusted for age, sex and wear time. Boys were more physically active than the girls. In terms of Total PA, boys were 2.75 min/h (95% CI [1.83–3.67], *p* < .001) more active, including 2.05 min/h (95% CI [1.37–2.73], *p* < 0.001) more MVPA than girls. There was a small, statistically significant effect of wear time in both models (Total PA model: 0.02 min/h, CI [0.01–0.02], p < 0.001*)*, indicating that children who were at the centre for longer periods tended to be more active per hour. Age was not a significant effect in either model.

## Discussion

This 22-week intervention, pragmatically designed to integrate physical activity into the daily routine of children attending childcare centres, produced a practically as well as statistically significant within-centre increase in Total PA and MVPA. Given the reported lack of effectiveness of pragmatic physical activity programs in childcare centres [6], the effect of our peer coaching approach on physical activity is a novel finding.

The practical significance is evident when considered alongside physical activity guidelines for preschool aged children. The current World Health Organization physical activity recommendation for preschool children is a minimum of 180 min/d, of which at least 60 min should be energetic play [23]. The effects of the AEL intervention group on Total PA and MVPA, at 28 min/d and 16 min/d respectively, corresponding to 23 and 27% of the WHO recommendations respectively, indicate that the peer-coaching approach made a substantial contribution towards children’s daily physical activity requirements.

The magnitude of the intervention effect is higher than previous multicomponent cluster RCT’s. For example, in the SPACE study involving 338 children across 22 centres, an 8 week intervention including resource provision and restructuring of outdoor time combined with a 4-h staff professional development session, resulted in a 1.3 min/h increase in MVPA and 2.2 min/h increase in Total PA. An important observation, and one requiring attention by future researchers, was that this effect was not maintained at 6-month post intervention, despite a booster professional development session at 4 months. The authors of another RCT, the SHAPES program [24], which involved 16 centres and 379 children, implemented a multicomponent program directed at training childcare educators to provide children with more opportunities to be physically active. They also reported a positive physical activity intervention effect, this time of 0.8 min/h. Recognising this as modest, the authors suggested that because it amounted to 35 min of MVPA per week, it was likely to benefit the health of high-risk youngsters, citing review evidence [25]. On the other hand, several evidence-informed and systematically designed interventions have not succeeded in improving physical activity. For example, the Jumpstart intervention [21] involved 658 children across 43 centres, a 7-h intensive workshop and a support visit to assist educators to implement a multicomponent approach to enhance physical activity. The authors concluded that low incidence of program implementation by the centre staff precluded any significant effect on physical activity. They also noted that ongoing professional development is likely to be critical for future success because it can take time for well-established settings to embed changes in routine. Another intervention unable to detect any effect on children’s physical activity was the 6 month Healthy Start-Départ Santé study, which studied 61 centres and 891 children, and involved a 3-h on-site training workshop for educators, provision of resources and active play equipment, and on-going on-line and telephone support [26].

To help explain the relatively greater magnitude of our intervention effect on physical activity, we can consider the likely influence of two program components. Common to most physical activity programs is professional development in some form, to assist educators to make changes to the childcare environment and/or their teaching practice. Several systematic reviews have indeed highlighted the importance of providing professional development because it targets educators who ultimately determine children’s daily routines, schedules and exposure to learning experiences relating to physical activity [9, 27]. In another recent systematic review it was suggested that programs which seek educator input and provide direct hands-on experience to build educator skills are those most likely to increase children’s physical activity [7]. These two aspects of professional development were indeed prominent in our current peer-coaching intervention and may have contributed to its strong effect on physical activity; and the central role of the peer coach warrants further discussion.

Peer-coaching directed professional development is a novel approach for physical activity intervention in the childcare centre setting. This approach was adopted in light of the promising results of a recently reported peer coaching physical activity intervention in primary schools [11]; together with review-based evidence suggesting its suitability to the childcare environment [28]. Directly applicable to our focus on physical activity in this report, the AEL peer-coaching approach facilitated the incorporation of three specific intervention elements previously identified as likely to lead to an increase in physical activity [7, 29]. These were the development of relationships, program flexibility, and a graduated integration of activities into the childcare daily routine; and we discuss these in turn below.

In developing beneficial relationships, our intervention drew on Vygotsky’s Social Development Theory which places emphasis on relationships between people (coach and childcare staff) within the sociocultural context (the childcare centre) in shared experiences (the activities). To this end the weekly 3-h site visits allowed the peer coach time to establish rapport and trust while working alongside the educators. Indeed, the coach’s diary indicated that the coaching strategy most frequently employed was to provide support for the educators (Fig. 2) by listening and attending to difficulties, directly or indirectly aimed at providing motivation toward implementation of the AEL program. The importance of the role of developing trustworthy and meaningful relationships between educators and interventionalist has been highlighted previously, especially when educators lack confidence and knowledge [30] and are asked to take on new responsibilities [29].

To facilitate program flexibility, the peer coaching approach was tailored to the individual needs of educators, providing freedom to determine when, how and where to implement the program activities throughout the week. This kind of tailored approach appears to be the strategy most consistently associated with improvements in children’s physical activity [7]. In the AEL program this was well facilitated by the weekly reflective and planning session between the AEL coach and educator which identified and addressed individual educator barriers for implementing the program. The previously mentioned SHAPES trial, which increased children’s physical activity, adopted a similar approach in allowing educators the flexibility to integrate physical activity opportunities in accordance with each centre’s unique features [24].

The gradual introduction of the AEL program to the educators was facilitated by the regular site visits, which enabled the coach to introduce activities at a rate commensurate with educator acceptance and capabilities. The program began with short and more easily implemented movement experiences during group times and transitions before progressing toward more structured activities requiring more planning and educator confidence. This method of professional development was informed by Vygotsky’s scaffolding approach in which the learner works in collaboration with an instructor to complete small, manageable steps in order to build confidence and competency to reach a goal. While success of the current program in enhancing physical activity suggests this approach has merit, further research is needed to understand the relative effectiveness of a peer-coach graduated introduction of content in comparison with other equally comprehensive but less continuous methods of professional development.

Finally, two general considerations may also assist in understanding the AEL program and help interpret its outcomes. The first is that it was informed by the concept of physical literacy, and so focussed on children’s enjoyment and movement competency. Daily activities within the group/mat time and the “transitional” activities were opportunistically directed towards improvement of fundamental movement skills and enjoyment, rather than an increase in physical activity per se. With the AEL coach always working with physical literacy in mind, together with the provision of the resource cards, educators were regularly reminded of the potential contribution of each activity to social, cognitive and physical development.

The second general consideration was that outcomes could be directly applied to real world childcare conditions. The AEL intervention featured a series of characteristics that support a pragmatic classification. Design processes took place in consultation with educators and managers; the peer coach employment was in line with industry standards; and research staff were removed from delivering the intervention. Therefore, the observed positive, clear effect on physical activity is in contrast with the conclusions from a metanalytic review which concluded that pragmatic physical activity interventions are typically ineffective in childcare settings [6]. On the other hand, any discussion of “real world” application is not complete without consideration of sustainability and as government policy makers and private childcare centre owners are aware, running costs are a primary concern. While our evidence suggests that a peer-coaching approach is likely to be successful in terms of improving physical activity, it involves appointment of a coach (the cost shared across 8 centres), which may be more expensive than other methods of professional development such as workshops and information sessions. Of practical economic importance would be further inquiry as to the optimal degree of peer-coaching required to exert an effect and sustain that effect on physical activity. Moreover, in a broader translational context, and considering the well-established health and developmental advantages of physical activity, a question arises requiring careful deliberation by childcare centre owners and government policy makers. “If you believe increasing physical activity and physical literacy is a desirable outcome in early childhood development, are you willing to allocate funding to support it?”

## Strengths and limitations

There are several strong aspects of this study, including the consultation with the centre owners and educators; access to centres with similar facilities, administration and staff qualifications; the RCT and statistical modelling and strongly significant findings; the theoretical underpinnings of the program; and the objective measure of physical activity. There are also several limitations of our work. The educator records of frequency of delivery of AEL activity components may have lacked accuracy (we trialled a chart to track activities, but discontinued when it appeared to compromise the coach-educator relationship); numbers of centres were limited by employment of the one coach and assessment resources; and the short window of opportunity for assessments adopted to minimise disruption to staff and children.

## Conclusion

The independent and direct benefits of physical activity to children’s physical and psychosocial development are well accepted, and with a large proportion of children enrolled in childcare centres, these services have an important role to play in providing programs which include sufficient physical activity. Using an RCT design, this study examined the impact of a peer-coached professional development intervention for childcare educators, designed to integrate physical activity into the daily curriculum. In contrast with previously conducted pragmatic interventions, the intervention elicited clear and strong practically significant effects on within-centre PA and MVPA, demonstrating the valuable contribution childcare centres may make to early physical activity.

## Data Availability

Data supporting the results reported in this article are stored at the University of Canberra. These data are available upon request by contacting the first author.

## Abbreviations

PA: Physical Activity
Total PA: Total Physical Activity
MVPA: Moderate to Vigorous Physical Activity
NQR: National Quality rating
SEIFA: Socio-Economic Index For Areas
RCT: Randomised Controlled Trial
WHO: World Health Organisation
